# A La_2_O_3_/MXene composite electrode for supercapacitors with improved capacitance and cycling performance

**DOI:** 10.1080/14686996.2023.2242262

**Published:** 2023-08-18

**Authors:** Jahangir Khan, Rana Tariq Mehmood Ahmad, Qiangmin Yu, Heming Liu, Usman Khan, Bilu Liu

**Affiliations:** a Shenzhen Geim Graphene Center, Tsinghua-Berkeley Shenzhen Institute & Institute of Materials Research, Tsinghua Shenzhen International Graduate School, Tsinghua University, Shenzhen, P. R. China; b Department of Electrical Engineering, Narowal Campus, University of Engineering and Technology, Lahore, Pakistan; c Institute of Functional Porous Materials, School of Materials Science and Engineering, Zhejiang Sci-Tech University, Hangzhou, P. R. China

**Keywords:** 2D materials, MXenes, lanthanum oxides, supercapacitors, electrode materials, capacitance, cycling

## Abstract

Developing efficient electrode materials is a key towards high power electrochemical energy storage devices. Two-dimensional (2D) MXene shows excellent conductivity and electrochemical performance among other materials. However, the restacking of MXene layers may degrade their specific capacity and cycling performance. Considering this challenge, here we have designed a composite made of 2D MXene nanosheets and lanthanum oxide (La_2_O_3_) nanoparticles to overcome the limitations. The bifunctionality of La_2_O_3_ nanoparticles prevents the restacking of MXene layers and enhances the electrochemical properties of the electrode due to its good Faradic characteristics. The specific capacitance of the La_2_O_3_/MXene composite electrode is 366 F/g at 1 A/g, which is 4.5 and 3 times higher than those of the individual La_2_O_3_ and MXene. The composite electrode displays a capacitance retention of 96% after 1,000 cycles, which is due to synergistic effects between the two components and indicates the potential of La_2_O_3_/MXene composite for supercapacitors.

## IMPACT STATEMENT

In this work, we designed a novel composite made of 2D MXene nanosheets and lanthanum oxide (La_2_O_3_) nanoparticles to enhance the supercapacitor performance. The excellent electrochemical performance stems from the synergistic effect between La_2_O_3_ nanoparticles and 2D MXene nanosheets of the composite electrode, where the La_2_O_3_ nanoparticles prevent MXene nanosheets from restacking, and MXene nanosheets offer fast charge transportation.

## GRAPHICAL ABSTRACT

**Figure:**
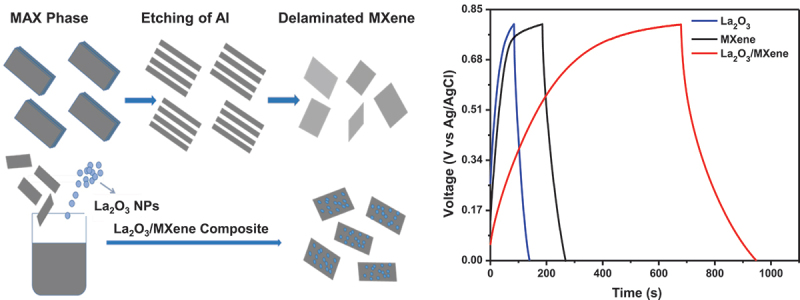

## 1. Introduction

The development of clean, portable, and efficient energy storage systems is essential as a consequence of rapid industrialization and the explosive growth of energy demands. The world is currently undergoing a severe energy crisis as a result of our overwhelming reliance on fossil fuels and other non-renewable energy sources [1–5]. Due to their intermittent nature, renewable energy sources are difficult to utilize because, when incorporated into electric grids, they cannot produce stable electricity continuously [6–11]. In order to reduce the rising demand for power globally, energy storage devices are important. These technologies not only satisfy the requirements for energy consumption in various fields but also additionally provide a sustainable and environmentally friendly alternative that can complement conventional energy sources [12,13]. In this regard, supercapacitors (SCs) and batteries play key roles among the various forms of energy storage technologies. The dominance of batteries in the market is owing to their high energy density; however, batteries show limited power output and require long-term use of energy [14,15]. Alternatively, SCs exhibit better performance in terms of their higher power density, larger specific capacitance, greater efficiency, and longer lifetime. Considering their above-mentioned features and their central role among energy storage devices, lots of efforts are ongoing to fabricate new electrode materials to further enhance SCs performance.

2D materials are promising electrode materials for high-performance SCs and other applications because of their intriguing properties, which include large specific surface areas, atomically thin 2D nature, mechanical flexibility, and physical and chemical properties [16–21]. MXenes refer to transition metal carbides, nitrides, and carbonitrides, belonging to the 2D material family that have drawn a lot of interest in SCs because of their excellent conductivity and hydrophilic nature [22–24]. Although MXenes exhibit excellent electrochemical properties, pending challenges, such as low capacitance, unreacted microstructures, and restacking of their layers, need to be addressed. To address these challenges, different strategies such as doping, surface modification, morphology control, and their hybrid composites with transition metal oxides (TMOs) have been applied to enhance the performance of MXene-based SCs [25].

In particular, significant progress has been accomplished in improving the electrochemical performance of SCs by integrating TMOs with MXenes [26]. However, the low electrical conductivity of TMOs hinders their application for high-performance SCs [27]. On the other hand, rare earth metal oxides have been explored for many potential applications because of their unique properties such as 4f electron configuration, trivalent oxidation state (+3), large intermolecular distance (8.45–8.70 Å), and anion-exchangeability [28]. Among various rare earth metal oxides, lanthanum oxide (La_2_O_3_) is considered as one of the most prominent candidates and has been utilized for many applications because of its low cost and excellent redox reversibility. La_2_O_3_ also exhibits good electrochemical properties such as excellent pseudocapacitance properties because of the coexistence of La^2+^ and La^3+^ during the charging and discharging processes, which enhances the redox reactions [29]. However, La_2_O_3_ material suffers from one major limitation, i.e., low electrical conductivity due to its wide band gap. Previous studies have shown that the SC performance of La_2_O_3_ materials can be improved by the formation of composites. For instance, Zhang et al. have synthesized MnO_2_/La_2_O_3_ composites by the hydrothermal method. The MnO_2_/La_2_O_3_ electrode material achieved a specific capacitance of 245 F/g at 0.3 A/g, which is larger than that of the individual La_2_O_3_-based electrode [30]. In another report, Sankar et al. designed a composite of La_2_O_3_ nanoparticles with rGO (La_2_O_3_/rGO), and the composite electrode material achieved a high energy density of 80 Wh kg^−1^ and a high power density of 2250 W kg^−1^ [31]. However, the achieved specific capacitance is still unsatisfactory for SCs applications and needs further improvements. Therefore, it is important to make La_2_O_3_/MXene composite with enhanced electrochemical performance for SCs.

In this work, we have designed an electrode material based on La_2_O_3_/MXene composite for the SC with improved capacitance and cycling performance by the synergistic effects between La_2_O_3_ and MXene. The La_2_O_3_/MXene composite shows high capacitance and stability when compared with MXene or La_2_O_3_ alone. The insertion of La_2_O_3_ (NPs) prevents MXene nanosheets from restacking, facilitating fast electron transfer and ion diffusion in the composite. The La_2_O_3_/MXene composite displays good electrochemical properties, including a high capacitance of 366 F/g at 1 A/g and excellent cyclic stability with a capacitance retention of 96% after 1,000 cycles. Our results suggest the effectiveness of such a strategy in improving the SC performance of 2D MXene materials.

## 2. Experimental section

### 2.1. Materials and chemicals

MAX powder (Ti_3_AlC_2_, 98% purity), hydrofluoric acid (HF, 40 wt%, Merck), dimethyl sulfoxide (DMSO, C_2_H_6_OS, 99%, Merck), lanthanum nitrate (La(NO_3_)_3_, 99.9%, Merck), NaOH, and polytetrafluoroethylene [(PTFE) (C_2_F_4_)_*n*_] membrane (pore size 100 nm) were purchased from Laisheng, Haining, China, and were used as received.

### 2.2. Preparation of MXene

The MXene nanosheets were produced by etching Al atoms from MAX structure (Ti_3_AlC_2_) powder following the method reported in the literature [32–34]. Briefly, 0.5 g Ti_3_AlC_2_ powder was added to the 10 mL of (40%) concentrated HF solution and stirred magnetically at 250 rpm for 24 h at room temperature. The resulting product was then centrifuged at 5,000 rpm and washed with deionized (DI) water to obtain MXene layer precipitates until the pH of the mixture reached 6. The resultant aqueous dispersions were subsequently vacuum-filtered using a PTFE membrane, and the obtained filtrate of MXene was subjected to freeze-drying for 24 h.

After adding 0.2 g of MXene powder to 15 mL DMSO, the mixture was magnetically stirred for 24 h at room temperature. The final product was centrifuged at 4,500 rpm for 15 min after being washed with DI water. To delaminate MXene flakes, the suspended scattered precipitate was ultrasonically treated for 1 h. The product was then centrifuged at 4,500 rpm for 30 min and heated in an oven at 70°C for 5 h.

### 2.3. Synthesis of La_2_O_3_ NPs

The La_2_O_3_ NPs were synthesized by following the reported method [35]. First, 25 mL 0.1 M La(NO_3_)_3_ aqueous solution was added into an aqueous solution of sodium hydroxide (25 mL, 1.0 M) to make a final volume of 50 mL. The obtained solution was then kept for stirring and refluxed at a moderate temperature for 2 h by using a magnetic stirrer. After stirring, the sample was centrifuged, washed with distilled water, and then dried at room temperature. The final product was then ground using mortar and pestle and transferred to calcinate at 300°C for 2 h.

### 2.4. Synthesis of the La_2_O_3_/MXene composite

The La_2_O_3_/MXene composite was made by combining 900 mg of La_2_O_3_ and 100 mg of MXene powder in 25 mL DI water and ultrasonically processed for 1 h. The resulting solution was then dried in a vacuum oven for 12 h at 75°C. The obtained nanostructures were ground to make a fine powder using a mortar and pestle [36].

### 2.5. Electrode fabrication

To investigate the electrochemical performance of SCs, three types of electrodes made of MXene, La_2_O_3_ NPs, and La_2_O_3_/MXene composite materials were fabricated. The preparation procedure of electrodes was described in the literature [36]. The active material (MXene, La_2_O_3_ NPs, and La_2_O_3_/MXene composite) was added to acetylene black and used as a binder in a mortar and pestle in an 8:1:1 ratio. To produce a thick slurry, a few drops of ethanol were added to the amalgam product that had been formed. The as-prepared slurry was then pasted on the single conducting side of a carbon cloth with an area of 1 × 2 cm^2^. The electrodes were then subjected to drying in the oven at 60°C. The weight of the applied material loading on the electrode surface is about 1–1.3 mg.

### 2.6. Material characterization

The phase and structural analyses of La_2_O_3_, MXene, and La_2_O_3_/MXene composites were performed by X-ray diffraction (XRD, JDX-352-JEOL, Tokyo, Japan, using Cu-Kα rays). The morphology and microstructure of the samples were characterized by scanning electron microscopy (SEM; JEOL SM6490, and Hitachi SU8010, 20 kV). Raman spectra were collected (LabRAM HR800, Kyoto, Japan) with a continuous-wave, frequency-doubled ND:YAG laser with a wavelength of 532 nm. The electrochemical measurements, such as charge transfer, C_sp_, and resistance, were done using cyclic voltammetry (CV), galvanic charge-discharge (GCD), and electrochemical impedance spectroscopy (EIS) techniques (Autolab PGSTAT12, USA). A three-electrode system with 0.1 M KOH as an electrolyte was used to perform the above mentioned measurements. MXene, La_2_O_3_, and La_2_O_3_/MXene were used as the working electrodes, platinum wire served as the counter electrodes, and Ag/AgCl served as the reference electrode.

## 3. Results and discussion

**Figure 1.**
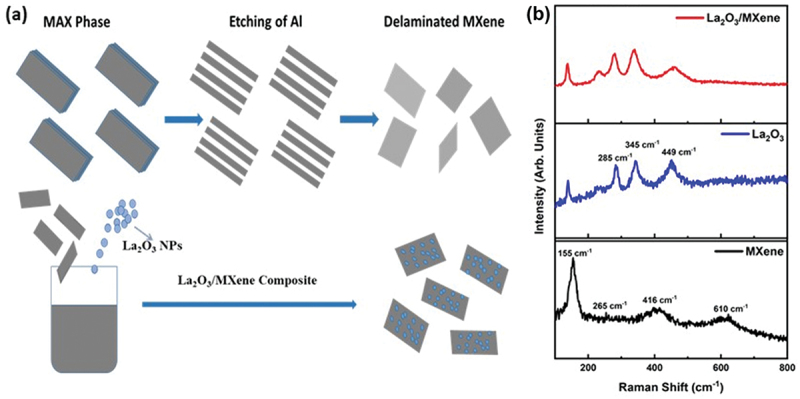
(a) Schematic of the synthesis process of the La_2_O_3_/MXene composite. (b) Raman spectra of MXene, La_2_O_3_ NPs, and La_2_O_3_/MXene composite.

We used a two-step co-precipitation method to prepare the La_2_O_3_/MXene composite (see details in the Experimental section). The combination of 2D MXene and La_2_O_3_ NPs may achieve synergistic effects in which MXene offers high electrical transport and a large surface area, and the electrochemically active La_2_O_3_ prevents MXene from restacking. Therefore, MXene and La_2_O_3_ may reinforce the performance of SCs through fast ion transportation and increased ion accessibility due to large interlayer spacing. The schematic of the synthesis of La_2_O_3_/MXene composite is shown in Figure 1(a). We used Raman spectroscopy to study the quality of the material and its structure. A comparison of MXene, La_2_O_3,_ and La_2_O_3_/MXene can be seen in Figure 1(b). The four Raman peaks at 155, 265, 416, and 610 cm^−1^ belong to MXene. These peaks are considered as a key feature of Ti_3_C_2_ and matched the literature [37,38]. Furthermore, the broadening of Raman peaks in the MXene spectrum indicates a decrement in the structural order due to the etching of Al atoms during the exfoliation process. The peaks at 300–700 cm^−1^ are typical bands related to the Ti-C bonding. For La_2_O_3_, three main peaks at 285, 345, and 449 cm^−1^ are assigned to the typical bands of the La_2_O_3_ structure and match the literature [39,40]. The peak located at 449 cm^−1^ corresponds to La-O vibrations. Further, the Raman spectrum of the La_2_O_3_/MXene composite shows the presence of peaks from both materials, indicating the formation of the composite.

**Figure 2.**
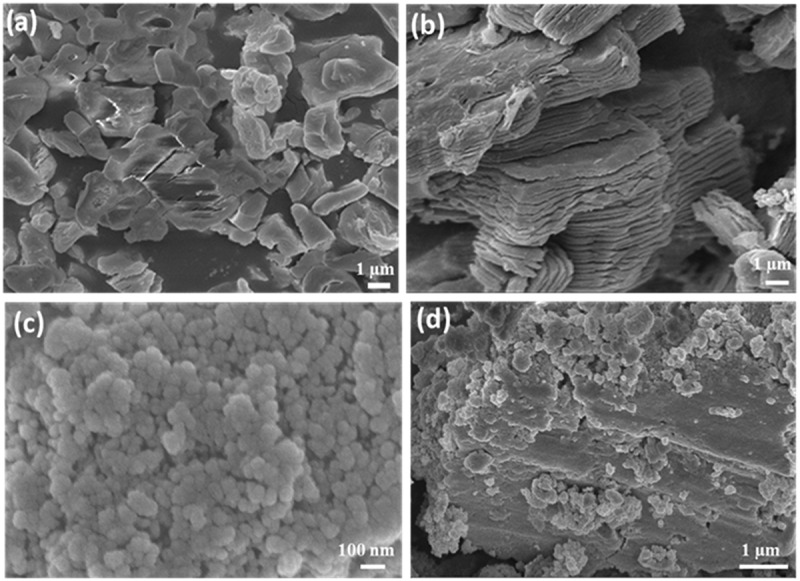
SEM images of (a) Bulk MAX, (b) MXene with exfoliated layers, (c) La_2_O_3_ NPs with non-uniform sizes, and (d) the La_2_O_3_/MXene composite.

SEM characterization was performed to study the surface morphology of MAX, MXene, La_2_O_3_ NPs, and La_2_O_3_/MXene composite. The bulk MAX layers are held tightly together due to the presence of Al atoms, as shown in Figure 2(a). After HF treatment of MAX, the elimination of Al atoms results in the exfoliation of layered MXene into 2D sheets, as illustrated in Figure 2(b). The accordion-like obtained structure of MXene was possibly due to an exothermic reaction between MAX and HF [41,42]. By further comparing the obtained results with the literature, it was confirmed that the 2D MXene nanosheets were synthesized successfully after HF treatment [43–47]. The statistical analysis of the La_2_O_3_ NPs (Figure 2(c)) shows that the La_2_O_3_ NPs are non-uniform in size with an average size of ~47 nm (Figure S1). The formation of the La_2_O_3_/MXene composite is confirmed by SEM images, as shown in Figure 2(d). The La_2_O_3_ NPs are mainly loaded on the surface of MXene and some small NPs are inserted into the layers to prevent MXene from restacking. Hence, the decoration of La_2_O_3_ NPs with MXene confirms the formation of the La_2_O_3_/MXene composite.

**Figure 3.**
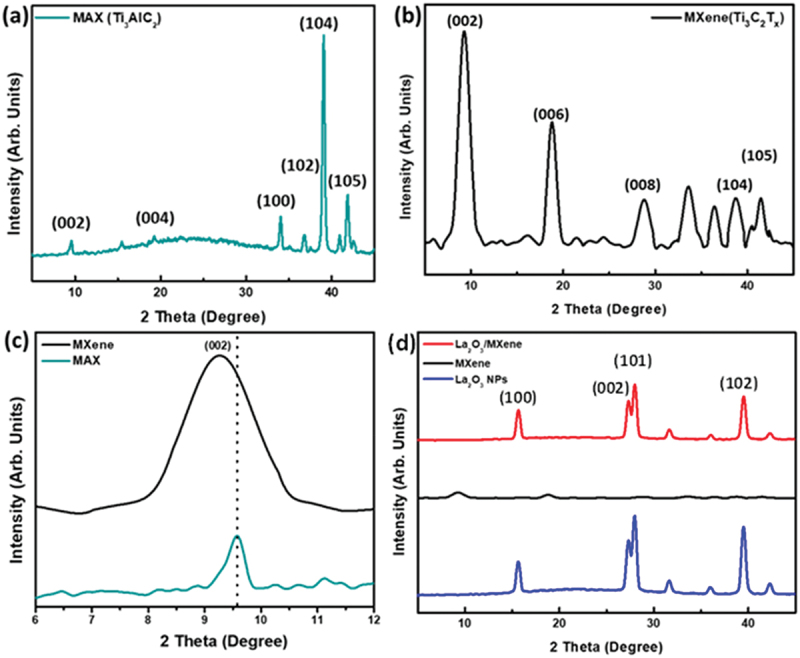
XRD patterns showing (a) MAX, (b) MXene, and (c) magnified XRD curves of MAX and MXene to show the peak shift. (d) XRD pattern comparisons of La_2_O_3_, MXene, and La_2_O_3_/MXene composite.

The phase of the as-synthesized MAX, MXene, and the La_2_O_3_/MXene composite was evaluated by XRD. The XRD patterns of pure Ti_3_AlC_2_ powder can be seen in Figure 3(a). For Ti_3_AlC_2_, a sharp characteristic (104) peak at 39° can be observed, which indicates the presence of Al atoms, along with other peaks at 41.7° (105), 36° (102), and 33.9° (100) showing the TiC impurity. The obtained XRD peaks of the Ti_3_AlC_2_ were indexed following the literature [48]. The XRD pattern of pure MXene (Figure 3(b)) shows a much decreased intensity of the characteristic (104) peak at 39° compared with MAX phase, due to the elimination of Al atoms from MAX sheets (Ti_3_AlC_2_) by using HF etchant. The XRD analysis further confirms that the characteristic peak of MAX becomes weaker, and broader as the HF reaction with MAX powder progresses, which results from a gradual decrement in the degree of the orderliness of planes. Figure 3(c) shows the magnified XRD pattern with a backward shift of (002) peak. One possible reason for this change may be the increase in the c-lattice parameter (LPc), where LPc = *d* spacing + thickness of 2D sheet, that ascribes the layered structure of MXene [49]. The XRD comparison of the La_2_O_3_, MXene, and La_2_O_3_/MXene composites is shown in Figure 3(d). It can be seen that the diffraction peaks of MXene became weak in the La_2_O_3_/MXene composite compared with La_2_O_3_ NPs due to the small amount of MXene used in the composite as well as the strong peaks from La_2_O_3_. The diffraction peaks at 15.70°, 27.40°, 29.60°, and 39.45° correspond to the (100), (002), (101), and (102) indices, respectively, which belong to La_2_O_3_ NPs. It agrees with the literature and confirms the successful synthesis of La_2_O_3_ NPs [50,51]. These results show that La_2_O_3_ NPs have been successfully grown on the MXene surface.

**Figure 4.**
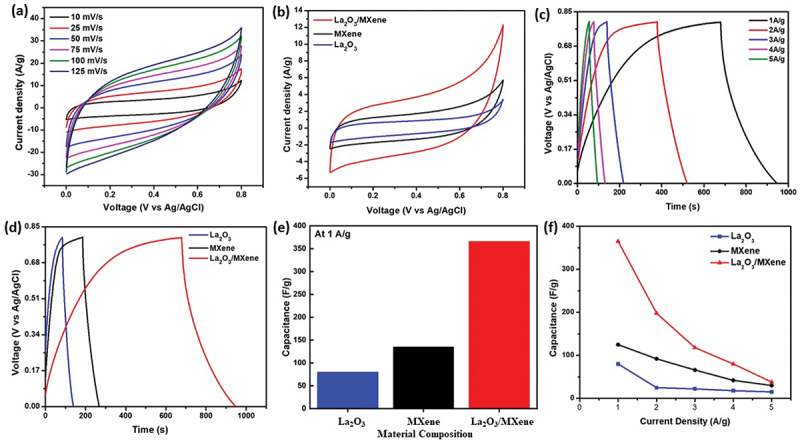
Supercapacitor performances. (a) CV loops of La_2_O_3_/MXene composite at different scan rates, (b) comparison of CV loop areas of La_2_O_3_, MXene, and La_2_O_3_/MXene composite at a scan rate of 10 mV/s, (c) GCD plots of La_2_O_3_/MXene composite at different current densities, (d) comparison of GCD plots of La_2_O_3_, MXene, and La_2_O_3_/MXene composite, (e) bar plots for the comparison of specific capacitance of La_2_O_3_, MXene, and La_2_O_3_/MXene composite, and (f) comparison or specific capacitance of three electrodes at different current densities.

The charge transfer rate at the electrode interface and the electrolyte are the key parameters to evaluate the electrochemical performance of SCs, which can be measured by CV. The cathodic and anodic peaks of MXene, La_2_O_3_, and the La_2_O_3_/MXene composite-based electrodes can be seen in Figure 4(a,b). Here, the cathode peaks are also known as reduction peaks in the negative current region, while the anodic oxidation peaks correspond to the positive current region. The variations in the CV loop areas at different scan rates of 10, 25, 50, 75, 100, and 125 mV/s were investigated under the same potential window ranges from 0 V to 0.8 V as illustrated in Figure 4(a). The results revealed that the areas of CV loops increased as the scan rates increased in all electrode materials. The possible reason is the large current that passes through the circuit at a large scan rate, ultimately increasing the loop area and C_sp_ value [52]. Furthermore, to analyze the variation of capacitance of MXene, La_2_O_3_, and La_2_O_3_/MXene-based composite, CV measurements were performed at a scan rate of 10 mV/s. The non-rectangular shapes of CV curves are clear evidence of Faradic behaviors exhibited by the electrode material during the whole C_sp_. The CV loop area of the composite-based electrode was greater than that of MXene and La_2_O_3_ alone, showing a large C_sp_ of La_2_O_3_/MXene composite-based electrode, as seen in Figure 4(b). The variations in the loop’s area might be attributed to the excellent conductivity and large surface area offered by the La_2_O_3_/MXene-based composite. The highly conductive MXene offered high charge carriers resulting in a decrement in internal resistance. Moreover, the large C_sp_ of the composite was achieved due to the insertion of La_2_O_3_ NPs in MXene, which provides improved Faradic behavior along with the electrical double-layer capacitance of MXene layers. Furthermore, the results obtained from CV loops of La_2_O_3_ and MXene at various scan rates can be seen in Figure S2 in the Supporting Information. The following formula can calculate the specific capacitance of the samples.

(1) $$C_{sp}=\frac{\int Idv}{vx\;\Delta Vxm}$$

Here, C_sp_ is the specific capacitance, ∆V is the voltage window, v is the scan rate, and ∫Idv is the area of the CV curve. Using the above formula, the calculated C_sp_ values of La_2_O_3_, MXene, and La_2_O_3_/MXene composite-based electrodes are 71, 125, and 298 F/g, respectively, at a scan rate of 10 mV/s. The La_2_O_3_/MXene composite electrode also showed excellent mechanical and structural stability with a capacitance retention of around 96% at 1,000 cycles (Figure S3). Furthermore, capacitance by CV loops at different scan rates was measured, and the bar plot of La_2_O_3_, MXene, and La_2_O_3_/MXene composite showing the best performance of composite-based electrode (Figure S4).

To further explore the electrochemical performance of the as-prepared electrode materials, GCD measurements which can evaluate the C_sp_ of SCs were performed at a current density of 1 A/g. Furthermore, variable current densities ranging from 1 A/g to 5 A/g were applied to investigate the effect of variable current densities on C_sp._ Increasing the current density decreases the area during the charge-discharge process, as shown in Figure 4(c). This effect is due to the redox reaction slowing down at high current density. In addition, at low current density, the electrolytic ions not only have a high probability of being adsorbed on the electrode surface but also have a high chance of penetrating inside the electrode. By further enhancing the current density value, the penetrating chances of ion charges reduce, and they can only reside on the outer surface of the electrode, which decreases the C_sp_ value of electrode materials [53]. C_sp_ of as-prepared samples is calculated by using the following formula [54]

(2) $$C_{sp}=2I/m\times \int Vdt/{\left(\Delta V\right)}^{2}$$

**Figure 5.**
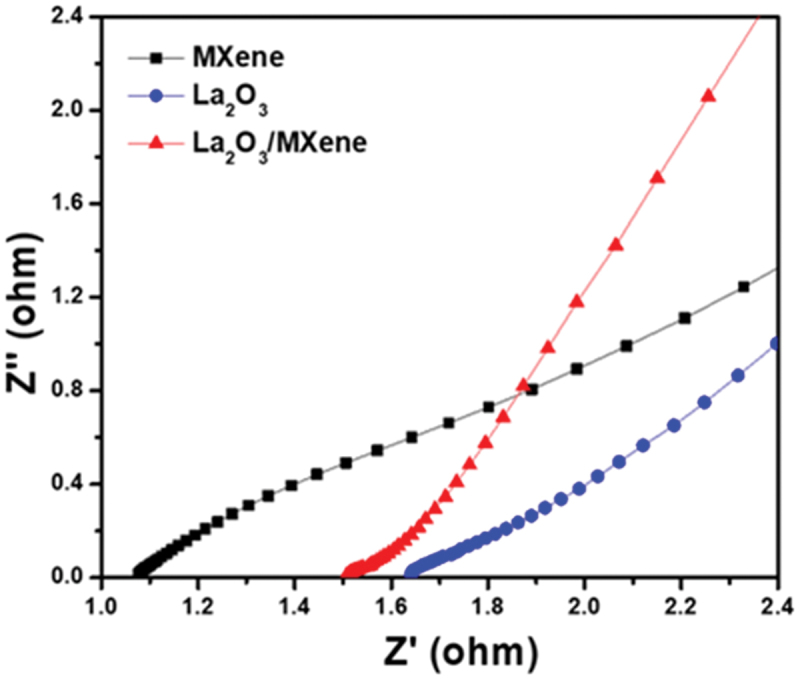
EIS curves of La_2_O_3_, MXene, and La_2_O_3_/MXene composite, showing internal charge transfer resistance of the La_2_O_3_, MXene, and La_2_O_3_/MXene composite.

Here, I/m is the current density (A/g), ∫Vdt is the area under the discharge curve, and ΔV is the potential window. The comparison of the GCD profiles of MXene, La_2_O_3_, and La_2_O_3_/MXene composite further revealed that the C_sp_ value of the composite is greater than that of the other two electrodes (Figure 4d). The obtained C_sp_ values of La_2_O_3_, MXene, and La_2_O_3_/MXene composite were 80, 135, and 366 F/g at 1A/g, respectively. The bar plots in Figure 4(e) show that La_2_O_3_/MXene composite has the best performance when compared with La_2_O_3_ and MXene individually. Furthermore, Figure 4(f) reveals that the C_sp_ decreases gradually with increasing the current density. The electrochemical performance of La_2_O_3_/MXene composite-based electrodes is better than some of the reported electrodes using rare earth oxide or MXene-based composite material, as illustrated in Table 1. The calculated GCD analysis of La_2_O_3_ and MXene can be seen in Figure S5. EIS was performed to evaluate the electrical resistance and charge transport properties of the electrode materials. EIS provides information about the internal charge transfer resistance of electrodes and the resistance between electrodes and electrolytes. Figure 5 shows the EIS curves of the MXene, La_2_O_3_, and La_2_O_3_/MXene composite under the frequency range of 100 kHz to 0.1 Hz with a Nyquist plot. Here, Zʹ and Zʹʹ correspond to the real and imaginary parts of impedances. The EIS result shows a linear line at a low frequency and a semicircle at a high frequency. The change in the shape and diameter of these semicircles depends on the transfer of charges at the electrode materials and ion’s movements [65,66]. The obtained charge transfer resistance of the La_2_O_3_, MXene, and La_2_O_3_/MXene composite were 1.7, 0.5, and 1.5 Ω, respectively, as given in Table S1. The composite material showed higher charge transfer resistance than MXene, which is attributed to the addition of semiconducting La_2_O_3_ NPs (Figure 5). The La_2_O_3_/MXene composite-based electrode exhibits good electrochemical performance because of combining highly conductive MXene with La_2_O_3_ NPs. The comparative study of C_sp_ obtained from CV, GCD, and charge transfer resistance is shown in Table S1.

**Table 1.** Performance comparisons of SCs made of La_2_O_3_/MXene composite electrode and literature using rare earth metal oxide or MXene-based composite.

Number	Materials	Synthesis Methods	Specific Capacitances (F/g)	References
1	La_2_O_3_/graphene	Reflux	156.2	[55]
2	MnO_2_/La_2_O_3_	Hydrothermal	245.4	[56]
3	La_2_O_3_ doped NiO	Hydrothermal	253.0	[57]
4	MnO_2_/MXene/CNT	Hydrothermal	181.8	[58]
5	MXene/MnO_2_/polyaniline	Hydrothermal	216.0	[59]
6	MXene/CNTs	Vacuum filtration	3.0	[60]
7	RuO_2_/MXene/CNF	Electrospinning	322.0	[61]
8	PANI/MXene	In situ polymerization Hydrothermal	337.5	[62]
9	MoO_3_/MXene@CC	Hydrothermal	442.0	[63]
10	Fe_2_O_3_/MXene	Hydrothermal	486.0	[64]
11	**La**_**2**_**O**_**3**_**/Ti**_**3**_**C**_**2**_**T**_***x***_	**Co-precipitation**	**366.0**	**This work**

## 4. Conclusion

In this work, we have developed a novel La_2_O_3_/MXene composite electrode for supercapacitors synthesized by a simple co-precipitation method. We find that the La_2_O_3_/MXene composite exhibits superior electrochemical performance compared to individual La_2_O_3_ and MXene electrodes. The obtained C_sp_ of La_2_O_3_, MXene, and La_2_O_3_/MXene composite from GCD results are 80, 135, and 366 F/g at 1A/g, respectively. The EIS analysis confirms the excellent electrochemical performance of La_2_O_3_/MXene composite with a small charge transfer resistance of around 1.5 Ω. Furthermore, the La_2_O_3_/MXene composite exhibits good mechanical and structural stability by attaining a capacitance retention of about 96% after 1,000 cycles. The synergistic effect between La_2_O_3_ NPs and 2D MXene layers boosts the electrochemical performance of the composite electrode material as the La_2_O_3_ NPs prevent MXene from restacking, and MXene offers fast charge transportation. The design strategy reported in this work may be extended to fabricate other MXene-based high-performance supercapacitors.

## Supplementary Material

Supplemental Material
