# Supplementary material for: A La2O3/MXene composite electrode for supercapacitors with improved capacitance and cycling performance
Source: Sci Technol Adv Mater. 2023 Aug 18;24(1):2242262. doi: 10.1080/14686996.2023.2242262 (PMC10443969; doi:10.1080/14686996.2023.2242262)
Supplement: Supplemental Material [file TSTA_A_2242262_SM1947.docx]

**Supplementary information**

**A La_2_O_3_/MXene composite electrode for supercapacitors with improved capacitance and cycling performance**

Jahangir Khan^†,^ ^‡^, Rana Tariq Mehmood Ahmad^‡^, Qiangmin Yu^†^, Heming Liu^†^, Usman Khan^#^, and Bilu Liu^*, †^

^†^Shenzhen Geim Graphene Center, Tsinghua-Berkeley Shenzhen Institute & Institute of Materials Research, Tsinghua Shenzhen International Graduate School, Tsinghua University, Shenzhen 518055, P. R. China.

^‡^ Department of Electrical Engineering, Narowal Campus, University of Engineering and

Technology, Lahore, Pakistan.

^#^Institute of Functional Porous Materials, School of Materials Science and Engineering, Zhejiang Sci-Tech University, Hangzhou 310018, P. R. China.

Correspondence should be addressed to [bilu.liu@sz.tsinghua.edu.cn](mailto:bilu.liu@sz.tsinghua.edu.cn) (B. L.)

**Figure S1.** Statistical analysis indicating La_2_O_3_ NPs size distribution with an average size of 47 nm.

**Figure S2.** CV loop curves of (a) La_2_O_3_ and (b) MXene at different scan rates.

**Figure S3.** CV loop curves of La_2_O_3_/MXene composite electrode at 1, 500, and 1,000^th^ cycle with a retention of 96% after 1000 cycles.


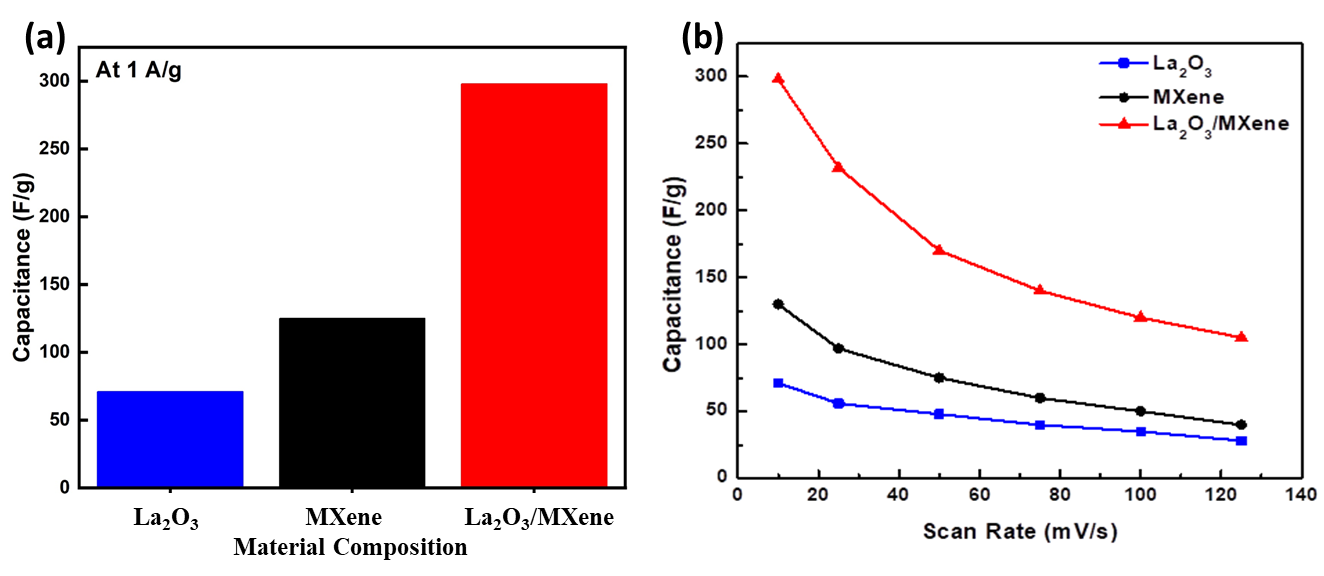


**Figure S4.** a) Bar plot for the comparison of specific capacitance of La_2_O_3_, MXene, and La_2_O_3_/MXene composite electrode obtained from CV loops. b) Comparison of specific capacitances at different scan rates.

**Figure S5.** GCD curves of a) La_2_O_3_ and b) MXene at different current densities.

**Table S1.** The comparative C_sp_ values were obtained from both CV and GCD along with charge transfer resistances of La_2_O_3_, MXene, and La_2_O_3_/MXene composite.

| Sr | Materials | Specific Capacitance  from CV (F/g) | Specific Capacitance  from GCD (F/g) | Charge Transfer Resistance (Ohm) |
| --- | --- | --- | --- | --- |
| 1 | La_2_O_3_ | 71 | 80 | 1.7 |
| 2 | MXene | 125 | 135 | 0.5 |
| 3 | La_2_O_3_/MXene | 298 | 366 | 1.5 |
